# Regulation of Molecular Biomarkers Associated with the Progression of Prostate Cancer

**DOI:** 10.3390/ijms25084171

**Published:** 2024-04-10

**Authors:** Miguel Martin-Caraballo

**Affiliations:** Department of Pharmaceutical Sciences, School of Pharmacy, University of Maryland Eastern Shore, Princess Anne, MD 21853, USA; mmartin@umes.edu

**Keywords:** prostate cancer, neuroendocrine differentiation, castration-resistant prostate cancer, interleukin-6, signaling, biomarkers

## Abstract

Androgen receptor signaling regulates the normal and pathological growth of the prostate. In particular, the growth and survival of prostate cancer cells is initially dependent on androgen receptor signaling. Exposure to androgen deprivation therapy leads to the development of castration-resistant prostate cancer. There is a multitude of molecular and cellular changes that occur in prostate tumor cells, including the expression of neuroendocrine features and various biomarkers, which promotes the switch of cancer cells to androgen-independent growth. These biomarkers include transcription factors (TP53, REST, BRN2, INSM1, c-Myc), signaling molecules (PTEN, Aurora kinases, retinoblastoma tumor suppressor, calcium-binding proteins), and receptors (glucocorticoid, androgen receptor-variant 7), among others. It is believed that genetic modifications, therapeutic treatments, and changes in the tumor microenvironment are contributing factors to the progression of prostate cancers with significant heterogeneity in their phenotypic characteristics. However, it is not well understood how these phenotypic characteristics and molecular modifications arise under specific treatment conditions. In this work, we summarize some of the most important molecular changes associated with the progression of prostate cancers and we describe some of the factors involved in these cellular processes.

## 1. Introduction

**Figure 1:**
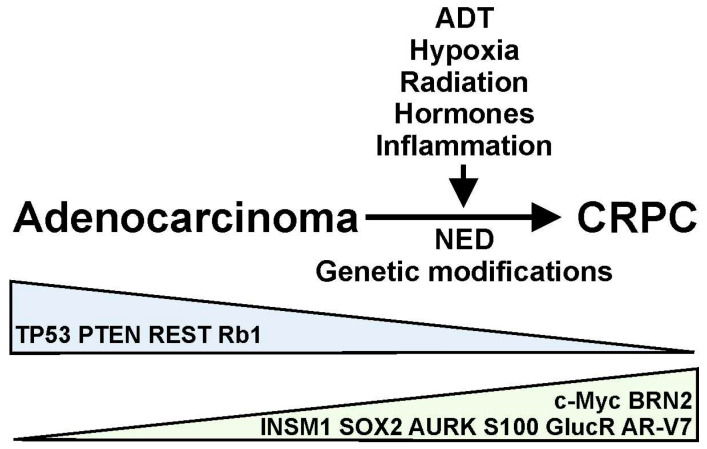
Androgen deprivation therapy (ADT), hypoxia, radiation, hormones, inflammation, and other factors can regulate the progression of prostate adenocarcinomas to castration-resistant prostate cancer (CRPC) through the induction of neuroendocrine differentiation (NED) and genetic modifications in prostate tumors. This progression results in the upregulation (green) or downregulation (blue) of various molecular markers, including transcription factors (TP53, REST, BRN2, INSM1, c-Myc) and cell signaling and receptor molecules (PTEN, Aurora kinases, retinoblastoma tumor suppressor, S100 proteins, CasR, Protein S, glucocorticoid receptor, AR-V7) in the prostate cancer cells.

Prostate cancer (PCa) is the most frequently diagnosed type of cancer and the second leading cause of cancer-related death among men [1,2]. The most common form of PCa in men is adenocarcinoma (>90%), characterized by an elevated production of prostate-specific antigen (PSA). The growth of PCa is initially androgen-dependent [3]. Androgen deprivation therapies (ADTs) are usually effective in causing tumor regression. Androgen receptors (ARs) are nuclear receptors that bind to testosterone and other androgens [4]. In the prostate, testosterone is converted to 5a-dihydrotestosterone (DHT) by the enzyme 5α reductase. DHT has a higher affinity for the ARs than testosterone [5]. The binding of testosterone or DHT to the ARs results in the formation of a complex that can migrate to the nucleus in order to regulate the expression of androgen-inducible genes [4]. However, prolonged exposure to ADT results in the progression of prostate tumors to a castration-resistant prostate cancer (CRPC) with a poor clinical prognosis (Figure 1). We should be aware that ADT does not result in a complete elimination of circulating androgens [6]. Furthermore, ADT has no effect on the levels of adrenal gland-produced androgens [7]. The molecular and cellular mechanisms involved in the transition of PCa to a castration-resistant phenotype are currently being explored in order to design better therapeutic treatments.

Both AR-dependent and AR-independent mechanisms have been proposed to regulate the resistance of PCa to ADT, resulting in the development of CRPC [3]. Several processes, including AR amplification and overexpression, AR mutations, and the expression of spliced variants, have been proposed to mediate AR-dependent resistance to ADT [3,8]. On the contrary, glucocorticoid receptor activation, immune-related processes, and neuroendocrine (NE) differentiation mediate the AR-independent resistance of PCa to ADT [3]. These various mechanisms can generate molecular changes in PCa, which results in the expression of a variety of biomarkers associated with disease progression (Figure 1). It is believed that genetic modifications, therapeutic treatments, and changes in the tumor microenvironment (TME) are contributing factors to the progression of PCa with variable phenotypic characteristics [9,10,11] (Figure 1). For example, ADT or radiation treatment of PCa patients can induce NE differentiation and the further progression of prostate tumors [3,12]. However, it is not well understood how these phenotypic characteristics and molecular modifications arise under specific treatment conditions. In this work, we summarize some of the most important molecular changes associated with the progression of PCa. More importantly, we describe some of the factors responsible for these cellular processes.

Trans-differentiation of PCa cells with NE features occurs in a subset of patients undergoing ADT. We should point out that a small number of NE cells are found in the normal prostate gland. However, ADT drives the appearance of foci of NE cells, leading to the formation of therapy-induced neuroendocrine prostate cancer (t-NEPC) [13,14]. It has been reported that approximately 20–25% of patients with CRPC show evidence of t-NEPC [15,16]. t-NEPC is an aggressive type of PCa with a poor prognosis that originates most likely from lineage plasticity. Based on its morphological features, NEPC can be divided into several sub-types, including prostate adenocarcinoma with NE differentiation, adenocarcinoma with Paneth cell NE differentiation, carcinoid tumor, small cell carcinoma (SCC), large cell NE carcinoma, and mixed NE carcinoma–acinar adenocarcinoma [17]. NE trans-differentiation of PCa cells shows higher expression of several neuronal markers, such as chromogranin, neuron-specific enolase (NSE), synaptophysin, and tubulin IIIβ [18,19,20]. However, the level of expression of individual neuronal markers shows significant heterogeneity among PCa tissue samples undergoing NE differentiation [21]. Both benign (found in the normal prostate) and malignant NE (found in NEPC) cells show low levels of expression of the AR and AR-downstream signaling [22]. Postmitotic NE cells secrete a variety of hormones and neurotransmitters capable of altering the mitogenic potential of the surrounding tissue [23,24]. Whether the appearance of NE cells following ADT is due to the transformation of multipotent progenitor cells or the trans-differentiation of prostatic adenocarcinoma cells remains to be determined [25,26]. However, single-cell analysis indicates that NE cells generated after ADT have an epithelial phenotype [27].

In vitro trans-differentiation of PCa cells, in particular LNCaP cells, can be reproduced via several mechanisms, including stimulation with cAMP-inducing conditions, interleukin-6 (IL-6), radiation, or hypoxia [28,29,30,31,32,33]. Culture of LNCaP cells with charcoal-stripped fetal bovine serum (FBS) can also be used to mimic androgen deprivation in vitro [34]. Similarly, increased cAMP signaling following receptor activation by hormones and drugs can promote NE trans-differentiation of PCa cells [35,36,37]. These factors can potentially play a role in the trans-differentiation of PCa in vivo. For example, inflammation and the resulting activation of immune cells can promote the release of various cytokines, contributing to the progression of PCa [9,10,38]. Changes in the TME can also promote tumor progression as a result of changes in the production of paracrine factors as well as the presence of a hypoxic environment [39,40].

ADT and the development of CRPC have been associated with the increased expression of several transcription factors, including TP53, REST, BRN2, and ISNM1, among others (Table 1, Figure 1). Whether the expression of these factors is regulated directly by the disruption of AR signaling or indirectly due to changes in the TME and the activation of various signaling mechanisms is not clearly understood (Table 1).

The tumor suppressor gene *TP53* encodes for the transcription factor p53 involved in cell cycle control and cell division. *TP53* loss of function is a common somatic alteration resulting in the development of CRPC [74]. It has been reported that ~50% of clinical samples of metastatic CRPC show several alterations in the *TP53* gene, including missense mutations, deletions, and gene truncations, which disrupt DNA binding [74,75]. Genomic alterations in *TP53* are commonly associated with the development of t-NEPC and metastatic PCa [74,76]. Loss of function of the *TP53* gene is a clinical predictor of increased resistance to abiraterone and enzalutamide in CRPC [77] and reduces the response of PCa cells to docetaxel [78]. The cellular and molecular mechanisms that give origin to the loss of function of *TP53* during the development of CRPC are not currently well understood. However, p53 function is associated with cellular stress, which may occur following treatment with ADT. In normal cells, p53 activity is low due to ubiquitination by the E3 ubiquitin ligase MDM2 [79]. Stress signaling results in increased activity of p53 and stimulation of gene transcription [41]. There is considerable cell proliferation in p53-null or mutated PCa cell lines (PC3, DU145) under hypoxic conditions, but not in LNCaP cells expressing wild-type p53 [42]. Culture of LNCaP cells with charcoal-stripped FBS-supplemented media evokes the induction of NED and a reduction in p53 expression, suggesting that androgen depletion promotes the loss of p53 function under these culture conditions [43]. The loss of function of p53 can also be related to an increased expression of negative regulators, such as MDMX and MDM2, which are highly expressed in PCa tumor samples derived from patients with CRPC [80] (reviewed in [81]).

The RE1-silencing transcription factor (REST) is a negative regulator of neuronal differentiation, repressing the expression of neuronal genes [82]. Downregulation of REST can be detected in approximately 50% of NE prostate tumors [83]. Proteomic analysis also reveals a significant reduction in REST expression in NEPC compared to prostate adenocarcinomas [84]. Molecular knockdown of REST expression results in a significant increase in the expression of NE markers, including chromogranin-B, secretagogin, and synaptophysin [83]. Overexpression of REST, on the contrary, downregulates the epithelial–mesenchymal transition (EMT), resulting in increased migration and invasiveness of prostate tumor cells [85]. In vitro experiments demonstrate a significant reduction in REST protein expression following the induction of NE differentiation of PCa cells with either IL-6 or hypoxia [44,45,46]. Hypoxia-induced downregulation of REST protein expression appears to involve proteasomal degradation [47]. The downregulation of REST expression following AR inhibition involves changes in CREB1 and PI3K/Akt signaling [48,86]. Thus, increased CREB1 activation by the beta receptor agonist isoproterenol downregulates REST expression in PCa cells, resulting in the induction of synaptophysin [86]. Similarly, both the molecular and pharmacological inhibition of PI3K/Akt signaling in LNCaP and PC3 cells downregulates REST expression and increases the expression of neuroendocrine biomarkers [48]. PI3K/Akt signaling regulates REST levels by promoting protein ubiquitination and degradation.

c-Myc (MYC) is a transcription factor belonging to the basic helix–loop–helix zipper class [87]. c-Myc acts as an oncoprotein associated with the progression of PCa to an androgen-independent phenotype due to its involvement in cell proliferation, growth, and differentiation. c-Myc mRNA expression increases severalfold following castration in rodents [88]. c-Myc mRNA and protein expression are also elevated in advanced PCa [89]. c-Myc expression drives proliferation in a PTEN/TP53-deficient mice model [49]. In vitro studies have shown that changes in c-Myc expression are mediated by external signals, including increased levels of IL-6 or androgen deprivation. For example, the stimulation of LNCaP cells with IL-6 for 6 days evokes a significant reduction in c-Myc expression [50,51]. Similarly, the exposure of LNCaP cells to androgen-depleted media (containing charcoal-stripped FBS, which mimics androgen depletion therapy in vitro) results in a significant reduction in c-Myc protein expression, indicating that both cytokines and androgen depletion contribute to c-Myc expression [51]. It has been demonstrated that AR activation directly regulates c-Myc transcription in a ligand-independent manner [90]. The overexpression of c-Myc in normal prostate luminal cells is sufficient to promote increased invasiveness in prostate tumors in vivo [91]. Other c-Myc family members, including N-Myc (MYCN) and L-Myc (MYCL), also appear to be differentially regulated based on the clinical state. For example, MYCN is highly expressed in CRPC [92] and MYCN gene amplification promotes the development of t-NEPC [93,94]. c-Myc interactions with various histone demethylases and regulatory factors have been identified in promoting the progression of PCa [51,95]. The direct interaction of c-Myc with the KDM4 family of histone lysine demethylases promotes the proliferation of CRPC [95], whereas c-Myc activation promotes the expression of the histone demethylases PHF8 and KDM3 [51]. c-Myc expression appears to be regulated by the loss of both *TP53* and *PTEN* expression [49]. Thus, in a double TP53- and PTEN-knockout mouse model, there is a significant increase in c-Myc expression due to the increased secretion of IL-6. This effect is not observed following the loss of *TP53* or *PTEN* separately.

The POU domain-containing transcription factor BRN2 also appears to be a critical driver of NE differentiation in PCa [96]. BRN2 proteins belong to a family of POU-containing transcription factors, involved in cell growth, cell cycle arrest, and differentiation [97]. There is a significant increase in BRN2 expression in NEPC compared to CRPC and prostatic adenocarcinomas [96]. BRN2 expression is regulated by the activation of ARs. Thus, there is increased BRN2 expression in enzalutamide-resistant prostate cancers, resulting in the aggressive growth of prostate cells in vivo and in vitro [96]. It appears that the upregulation of BRN2 expression following the development of enzalutamide resistance involves the pseudo-kinase Tribbles 2 (TRIB20) [52]. Short-interference RNA used against TRIB2 not only reduces the expression of the luminal cell markers AR and cytokeratin 8, but it also results in an increased expression of BRN2 [52]. BRN2 expression in neuroendocrine PCa is also under the regulation of the heterodimeric protein Mucin-1 (MUC1). It has been determined that MUC1 is overexpressed in prostate tumors [53]. MUC1 activation by Tyr kinases’ downstream signaling regulates various transcription factors. In LNCaP cells, the downregulation of MUC1 also results in a reduction in BRN2 expression [54].

Insulinoma-associated protein 1 (INSM1) is a zinc-finger transcription factor implicated in the NE of cancer cells [98,99]. INSM1 acts as a transcriptional repressor involved in cell cycle arrest [98]. By binding to cyclin D1, INSM1 promotes cell cycle arrest and inhibits cell proliferation [100]. A majority of SCCs, mixed SCC–acinar adenocarcinomas, and metastatic SCCs derived from prostate cancer patients are stained for INSM1 [101]. While neuroendocrine PCa cells express high levels of INSM1, benign prostate tissue lacks any expression of this protein, suggesting that INSM1 can be a specific marker of advanced PCa [101]. The functional role of INSM1 in highly proliferative PCa, such as in SCC, is still debatable.

It has been recognized that cell lineage plasticity and reprogramming in prostate tumors contribute to cancer progression following ADT [102]. The induction of t-NEPC causes the expression of pluripotential stem cell genes, such as the transcription factors LIN28B and SOX2 [103]. The SRY (sex-determining region Y) box 2 (SOX2) gene has been implicated in the development of CRPC [55]. In a normal prostate, SOX2 expression is limited to NE cells [56]. The SOX2 level is also low in primary adenocarcinomas of the prostate, but its expression is found in the majority of metastatic PCa patients, especially those characterized by NE features [56]. In vitro experiments indicate that SOX2 expression is low in LNCaP cells but increases significantly in androgen-independent PCa cells (PC3, DU145) [56,57]. Functionally, SOX2 expression upregulates genes associated with pluripotency and EMT, which promotes the migration and invasion of PCa cells [57]. In vitro and in vivo experiments indicate the presence of a transcriptional network regulating SOX2 expression via LIN28B function [103]. SOX2 expression is also repressed by AR activation [55]. Thus, pharmacological inhibition of AR function reverses the transcriptional repression of SOX2 expression and promotes the growth of CRPC [55]. SOX2 expression is also regulated by *TP53* [75]. Loss of function of both *TP53* and *Rb1* (see below) promotes lineage plasticity and resistance to ADT by increasing SOX2 expression [58]. However, we should point out that alterations in both *TP53* and Rb1 expression are not an absolute requirement for the development of NE PCa, indicating significant divergence in the molecular signature of NEPC [104].

## 2. Cell Signaling Molecules and Receptors: PTEN, Aurora Kinases, Retinoblastoma Tumor Suppressor, Glucocorticoid Receptor, AR-V7, and Others

Significant changes in signaling molecules and their underlying networks drive the progression of PCa to a castration-resistant phenotype (Table 1, Figure 1). The development of CRPC is often associated with a loss of function of the phosphatase and tensin homolog (PTEN) tumor suppressor. PTEN is a lipid phosphatase involved in the dephosphorylation of phosphoinositide 3,4,5-triphosphate (PIP3), a main substrate for Akt protein activation [105]. The phosphoinositide 3-kinase (PI3K) is responsible for PIP3 phosphorylation in response to growth factors or other signaling molecules. Akt activation regulates various targets involved in apoptosis and cell cycling, including BAD, caspase 9, and Bcl-2 [106,107]. PTEN deletions can be detected in ~40% of clinical samples derived from prostate tumors [75,108] and predict the efficacy of abiraterone and enzalutamide cancer treatments [108]. PTEN genomic alterations are also detected in metastatic PCa [74]. Functionally, the loss of PTEN function results in increased Akt/PI3K and Ras/ERK signaling, which promotes cell proliferation and migration [105]. In a PTEN-knockout mouse model, there is a significant increase in DNA GpG methylation and alterations in gene transcription associated with inflammatory and immune responses during progression of PCa [109]. PTEN expression is elevated only in the DU145 PCa cell line, but little expression is detected in LNCaP or PC3 cells [107,110], suggesting that the regulation of PTEN expression may occur independently of AR function. However, AR transcriptional activity is negatively regulated by PTEN expression [59]. The genetic mechanism(s) that results in the loss of function of PTEN during the development of CRPC are not well understood. However, several miRNAs are involved in downregulating PTEN expression in PCa, suggesting that the deregulation of miRNA expression mediates the loss of PTEN function during PCa progression [60].

Aurora kinases (AURKs) are a family of serine/threonine kinases involved in cell cycle regulation [111]. AURK-A regulates proper spindle formation and chromosome segregation during mitosis, whereas AURK-B regulates centrosome separation, chromosome segregation, and cytokinesis. Increased expression of AURKs has been associated with the progression of PCa to a castration-independent phenotype [61]. In vitro studies have shown that expression of both AUK-A and AUK-B RNA is significantly elevated in androgen-independent PCa cell lines (PC3, DU145) and low in androgen-dependent LNCaP cells [61]. AURK-A is also highly expressed in CRPC samples [61]. AUK-A and AUK-B are also elevated in clinical samples derived from prostate tumors when compared to samples derived from normal prostatic tissue [112]. Although AURK-A and AURK-B protein expression is undetectable in androgen-dependent LNCaP cells, immunoblot analysis shows significant expression in PC3 cells, a model for SCC [46,113]. In vitro studies have also demonstrated that IL-6 or cAMP-inducing agents do not regulate the expression of AURKs following the induction of NED [46,114]. AURK-A expression appears to be under the regulation of AR signaling. Thus, overexpression of the androgen receptor in LNCaP cells causes a significant increase in AURK-A transcripts [61]. Pharmacological inhibition of AURK activity or silencing of gene expression results in a significant reduction in PCa cell proliferation [61,113,115]. Furthermore, the downregulation of AURK-A function sensitizes PCa cells to further treatment with antimitotic agents, indicating a considerable potential of targeting AURK-A to limit PCa growth [115]. AURK expression is also regulated by the transcription factors c-Myc and REST [62,63]. In patients with metastatic CRPC, there is a direct relationship between c-Myc and AURK-A expression [116]. In t-NEPC, there is a co-amplification of AURK-A and N-Myc in ~70% of analyzed tumor samples [93]. Similarly, genomic profiling of REST-regulated genes indicates that siRNA-evoked downregulation of REST causes a significant decrease in AURK-A expression [63].

The retinoblastoma tumor suppressor protein (Rb1) regulates androgen-dependent cell proliferation [117]. Rb1 is a negative regulator of the cell cycle that is encoded by the human retinoblastoma gene. The regulation of Rb1 activity by phosphorylation occurs in a cell cycle-dependent manner. Low-level phosphorylation of Rb1 in quiescent cells inhibits cell cycle progression, whereas mitogenic signals promote Rb1 phosphorylation and cell division [118]. Genomic studies indicate that downregulation of Rb1 expression is correlated with advanced prostate cancer, in particular SCC [119,120]. In SCC, Rb1 loss is observed in ~90% of all samples analyzed, often co-occurring with the loss of *TP53* expression [16,119]. On the contrary, metastatic CRPC and acinar carcinomas with NED demonstrate little Rb1 loss [119]. Furthermore, the downregulation of Rb1 expression alters the response of PCa cells to therapeutic interventions [121]. Thus, Rb1 depletion in androgen-dependent LNCaP cells promote cell proliferation under conditions that mimic ADT [121]. We should point out that Rb1 and PTEN loss often co-occurs in neuroendocrine PCa [120,122]. Lower expression of Rb1, together with PTEN and *TP53*, is associated with shorter overall survival in patients receiving ADT and docetaxel combination treatment [123]. The loss of *TP53* and Rb1 also promotes resistance to ADT [58]. However, we should point out that alterations in both *TP53* and Rb1 expression are not an absolute requirement for the development of NE features, indicating significant divergency in the molecular signature of NEPC [104,124]. Regarding the mechanism of Rb1 loss in PCa cells, a significant reduction in Rb1 expression has been demonstrated following the induction of NE differentiation in LNCaP cells with IL-6 in vitro [46]. It appears that the downregulation of Rb1 function is also under the influence of AR activation. In vitro experiments have demonstrated that increased signaling via the Raf/MEK/ERK pathway results in a downregulation of AR expression and a reduction in Rb1 phosphorylation, promoting NED in LNCaP cells [64].

The progression of PCa to a castration-resistant phenotype is also accompanied by significant changes in calcium homeostasis and the associated proteins. S100 proteins are a large family of calcium-binding proteins involved in the regulation of cell proliferation, differentiation, and migration [125]. Several S100A proteins (S100A8, S100A9) have been identified as biomarkers for prostate tumors, exhibiting increased expression in CRPC [126,127]. Analysis of the transcriptome of LNCaP cells undergoing the progression to CRPC in vivo reveals a significant increase in S100A10 transcripts [128]. Tissue microarray analysis of CWR22 xenografts indicates a high level of expression of S100P [128]. S100P expression is upregulated through the stimulation of cultured LNCaP cells with IL-6 [129]. However, S100A9 protein expression does not appear to be regulated by 4-day stimulation with IL-6 or cAMP-inducing agents in vitro [46]. The expression of S100A9 in non-treated LNCaP or C4-2 PCa cell lines is non-existent and 4-day stimulation with IL-6 or cAMP-inducing agents has no significant effect on S100A9 protein levels. However, S100A9 protein expression is elevated in PC3 cells compared to LNCaP or C4-2 PCa cell lines [46]. On the contrary, long-term culture of LNCaP cells with charcoal-stripped FBS media evokes NED and an increased expression of S100 proteins [29]. Similarly, blocking AR signaling results in a significant increase in S100P expression [65]. These findings suggest that disruptions to androgen receptor signaling, IL-6, and cAMP signaling may have different roles in the regulation of S100 proteins.

The calcium-sensing receptor (CasR) is a G-protein surface receptor that interacts with various ligands, including calcium ions, vitamin D, and IL-6. CasR regulates calcium homeostasis in the human body by controlling the synthesis and secretion of the parathyroid hormone (PHT) [130]. CasR expression has been associated with the development of lethal PCa [131]. PCa metastasis in the bone expresses elevated levels of CasR compared with cancer tissue from the prostate [132]. CasR expression is regulated by vitamin D [66]. CasR transcripts and protein are highly expressed in PC3 cells, but not in LNCaP PCa cells [133,134]. Pharmacological inhibition of CasR activity prevents the development of NE features in PC3 and 22Rv1 cells in vitro [134]. Furthermore, 4-day stimulation of LNCaP cells with IL-6 or cAMP-inducing agents does not affect the expression of CasR in vitro [46].

Protein S (also known as PROS) is encoded by the PROS1 gene. The function of Protein S is often associated with anticoagulation activity. However, high levels of Protein S expression are detected in high-grade and metastatic PCa sample tissues, where it appears to regulate cell proliferation and migration in prostate tumors [135]. Proteomic analysis reveals an increased expression of Protein S in androgen-independent PCa cells (PC3, DU145) and low expression in LNCaP cells [136]. Immunoblot analysis also reveals a high expression of Protein S in PC3 cells, but not in LNCaP or C4-2 cells [46]. However, Protein S expression is not affected by the stimulation of PCa cells with IL-6 or cAMP-inducing agents. Thus, Protein S does not appear to be regulated by IL-6 or cAMP signaling in vitro.

Increased expression of the glucocorticoid receptor (GlucR) and AR-V7 has been implicated in the androgen-independent growth of PCa [137,138]. It has been proposed that GlucR activation by low-level circulating androgens can bypass androgen receptor blockage and stimulate PCa cell growth following ADT [137]. Clinically, increased GlucR expression in patients with CRPC promotes resistance to enzalutamide or abiraterone [139]. Furthermore, pharmacological inhibition of GlucR function reverses the resistance to docetaxel in both PCa patients and docetaxel-resistant PCa cell lines [140]. Upregulation of GlucR expression is associated with radiation resistance and progression to androgen independence in both LNCaP and C4-2 PCa cell lines [141]. It has been established that GlucR expression is significantly high in androgen-independent PCa cell lines (PC3, DU145) and negligible in androgen-dependent LNCaP cells [46,142]. Functionally, GlucR expression may regulate other molecules implicated in the NE differentiation of PCa. For example, GlucR knockdown also results in the transcriptional downregulation of the N-Myc transcription factor [143]. GlucR-evoked increases in N-Myc expression also promote Rb1 signaling, resulting in the expression of various NED markers in PCa cells [143]. The expression of GlucR is negatively regulated by AR signaling [67]. Thus, culture of LNCaP cells with AR blockers or charcoal-stripped FBS evokes the expression of GlucR transcripts [67]. However, GlucR protein expression is not regulated by the stimulation of LNCaP with IL-6, whereas cAMP-stimulating agents promote GlucR expression in vitro [46]. Thus, the upregulation of GlucR expression is under the control of AR and cAMP signaling.

Alterations in AR function as a result of genetic modifications or splicing also contribute to the progression of PCa [144]. In the normal prostate and the LNCaP cell line, the AR gene generates a protein with a molecular weight of ~110 kDa [145]. However, in CRPC and the 22RV1 cell line, an AR variant with an ~80 kDa molecular weight is also detected [144,145]. This spliced variant, called AR-V7, exhibits a constitutively ligand-independent activity that results in increased cell proliferation in an androgen-independent manner [144]. Ligand-independent activation of AR-V7 occurs due to truncation in the ligand-binding domain (LBD) of the wild protein. Genetic silencing of AR-V7 with siRNA or pharmacological inhibition of the AR protein with MDV3100 blocks cell growth [144]. Clinically, the increased expression of AR-V7 in bone metastasis is associated with resistance to AR and limited patient survival [146]. The transcriptional activity of AR-V7 is regulated by the PTEN-PI3K-Akt signaling pathway, since PTEN inactivation increases AR-V7 activity [68]. Regulation of AR-V7 expression appears to involve both transcriptional and post-translational mechanisms. ADT using MDV3100 or abiraterone treatment increases the expression of spliced variants, such as AR-V7, in a subset of CRPC tumors [69]. There is a positive correlation between AR-V7 and histone demethylase JMJD1A expression in prostate cancer specimens [70]. Histone demethylation via JMJD1A promotes both the mRNA and protein expression of AR-V7 [70]. AR-V7 protein expression is also regulated by the balance between protein kinase and phosphatase activity [71]. Thus, changes in the activity of phosphatase PP-1 and Akt result in the phosphorylation of AR-V7, leading to ubiquitination and degradation of the receptor protein [71]. In vitro experiments on the 22RV1 cell line also reveal that AR-V7 expression is regulated by NF-κB signaling [72]. Pharmacological inhibition of NF-κB signaling downregulates AR-V7 expression both in vivo and in vitro [72,73].

## 3. Conclusions

In conclusion, it appears that the progression of PCa results in significant molecular changes and the expression of various biomarkers (summarized in Table 1). This contributes to the heterogeneity of PCa seen at various stages of tumor development. In vitro and in vivo results point to various intrinsic and extrinsic factors in driving these changes. However, we need a better understanding of the cellular and molecular mechanisms involved in this transformation in order to design better treatments for prostate tumors.

## 4. Future Directions

Intrinsic and extrinsic factors regulate the progression of PCa cells, resulting in the heterogeneous expression of a multitude of molecular biomarkers. These molecules play a significant role in altering the survival, proliferation, and migration of PCa cells. A significant challenge to be addressed in future research is the assessment of how different treatment conditions alter the ability of prostate tumor cells to transition to a castration-resistant and/or neuroendocrine phenotype in vivo. Large-scale transcriptomic and genetic analyses are required to elucidate the effect of multiple factors in regulating molecular changes in prostate tumors. It will be particularly challenging to study how different interacting conditions contribute to the overall phenotype of advanced prostate tumors. This can lead to the development of more personalized treatments for advanced prostate tumors targeting specific signaling mechanisms.

## Figures and Tables

**Table 1:** Biomarkers associated with the molecular differentiation of prostate cancer.

Biomarker	Function	Regulatory Signaling	References
TP53	Transcription factor, tumor suppressor	Cellular stress, ARS	[41,42,43]
RE1-silencing transcription factor (REST)	Transcription factor, neuronal suppressor	IL-6, hypoxia, ARS, PI3K/Akt	[44,45,46,47,48]
c-Myc (MYC)	Basic helix–loop–helix zipper transcription factor	IL-6, loss of function of PTEN and TP53	[49,50,51]
BRN2	POU domain-containing transcription factor	Pseudo-kinase Tribbles 2, Mucin-1	[52,53,54]
Insulinoma-associated protein 1 (INSM1)	Zinc-finger transcription factor, transcriptional repressor	UNK	
SOX2	SRY (sex-determining region Y) box 2 transcription factor	ARS, TP53	[55,56,57,58]
Tensin homolog (PTEN)	PI3K phosphatase	ARS, miRNA	[59,60]
Aurora kinases (AURKs)	Serine/threonine kinase	ARS, c-Myc, REST	[61,62,63]
Retinoblastoma tumor suppressor protein (Rb1)	Cell cycle regulator, tumor suppressor	IL-6, ARS	[46,64]
S100 proteins	Secreted proteins	ARS	[29,46,65]
Calcium-sensing receptor (CasR)	G-protein-coupled receptor	VitD	[66]
Protein S (also known as PROS)	Plasma glycoprotein	UNK	
Glucocorticoid receptor (GlucR)	Intracellular receptor	ARS, cAMP	[46,67]
Androgen receptor variant 7 (AR-V7)	Intracellular receptor	ARS, PI3K/Akt, NF-κB	[68,69,70,71,72,73]

ARS: androgen receptor signaling evoked by androgen deprivation therapy in vivo or culture with cs-FBS in vitro; UNK: unknown regulatory signaling.

## Data Availability

Not applicable.

## Abbreviations

ADT	androgen deprivation therapy
AR	androgen receptor
CRPC	castration-resistant prostate cancer
DHT	dihydrotestosterone
EMT	epithelial-to-mesenchymal transition
FBS	fetal bovine serum
IL-6	interleukin-6
LNCaP	lymph node carcinoma of the prostate
NE	neuroendocrine
NED	neuroendocrine differentiation
NSE	neuron-specific enolase
PCa	prostate cancer
PSA	prostate-specific antigen
SCC	small cell carcinoma
TME	tumor microenvironment
t-NEPC	therapy-induced neuroendocrine prostate cancer
